# Buccal Absorption of Biopharmaceutics Classification System III Drugs: Formulation Approaches and Mechanistic Insights

**DOI:** 10.3390/pharmaceutics16121563

**Published:** 2024-12-06

**Authors:** Rayan Sabra, Daniel Kirby, Vikram Chouk, Kleta Malgorzata, Afzal R. Mohammed

**Affiliations:** 1 Aston Pharmacy School, Aston University, Birmingham B4 7ET, UKd.j.kirby1@aston.ac.uk (D.K.); 2 Catalent Pharma Solutions U.K. Swindon Zydis^®^ Limited, Swindon SN5 8RU, UK

**Keywords:** buccal absorption, BCS III drugs, permeability, formulation approaches, permeation enhancers

## Abstract

Buccal drug delivery emerges as a promising strategy to enhance the absorption of drugs classified under the Biopharmaceutics Classification System (BCS) Class III, characterized by high solubility and low permeability. However, addressing the absorption challenges of BCS Class III drugs necessitates innovative formulation strategies. This review delves into optimizing buccal drug delivery for BCS III drugs, focusing on various formulation approaches to improve absorption. Strategies such as permeation enhancers, mucoadhesive polymers, pH modifiers, ion pairing, and prodrugs are systematically explored for their potential to overcome challenges associated with BCS Class III drugs. The mechanistic insight into how these strategies influence drug absorption is discussed, providing a detailed understanding of their applicability. Furthermore, the review advocates for integrating conventional buccal dosage forms with these formulation approaches as a potential strategy to enhance absorption. By emphasizing bioavailability enhancement, this review contributes to a holistic understanding of optimizing buccal absorption for BCS Class III drugs, presenting a unified approach to overcome inherent limitations in their delivery.

## 1. Introduction

Oral drug delivery is the preferred and most widely used route of administration, owing to its convenience and non-invasive nature [1]. Nevertheless, certain drugs face challenges in achieving optimal absorption and bioavailability through this route due to their specific physicochemical properties. The Biopharmaceutics Classification System (BCS) plays a crucial role in classifying drugs based on their solubility and permeability characteristics, providing valuable insights into their behavior in the human body [2]. BCS categorizes drugs into four classes (I to IV) based on their solubility and permeability. BCS Class I drugs are both highly soluble and highly permeable, making them good candidates for oral drug delivery [3]. In contrast, BCS Class III drugs, which are predominantly hydrophilic with a poor permeability profile, present a significant challenge in achieving effective oral delivery. These drugs exhibit low bioavailability due to challenges in crossing biological membranes [4] and are primarily excreted unchanged in the bile and urine [5]. In this context, our focus will be on BCS Class III drugs, exploring the potential strategies to enhance their absorption.

Improving the absorption and bioavailability of BCS Class III drugs is essential for enhancing their therapeutic efficacy and reducing the required dose, potential side effects, and overall cost of treatment. A promising approach for enhancing the absorption of such drugs is through buccal delivery, where drugs are absorbed through the buccal mucosa before they reach the systemic circulation [6]. Buccal mucosa is a 0.5–0.8 mm [7] thick area located in the oral cavity between the gums and lower lips, with an average surface area of approximately 50 ± 2.9 cm^2^ in human adults [8]. The buccal routes offer several advantages over traditional oral administration. It bypasses first-pass metabolism, allowing the drug to be absorbed directly through the blood vessels into the systemic circulation, and provides a non-invasive and convenient mode of drug delivery [9]. For example, in a study by Pickering et al. [10], two randomized clinical trials were conducted to evaluate the efficacy of buccal acetaminophen for pain relief in healthy volunteers compared to sublingual and intravenous routes. The authors found that buccal administration of acetaminophen provided faster analgesia, with an onset of pain relief within just 15 min, compared to other routes, and this rapid effect may be attributed to both the physicochemical properties of the pharmaceutical form and the unique physiology of the buccal mucosa. As such, they suggest that buccal administration is a better alternative to other routes of administration as it produces a faster onset of action [10]. Furthermore, drugs administered through this route may exhibit enhanced stability, owing to the mouth’s relatively neutral pH in contrast to the varying pH levels present in other parts of the gastrointestinal system [8].

However, buccal administration comes with challenges due to the unique characteristics of the buccal mucosa, including the presence of enzymes like aminopeptidase, carboxypeptidase, and esterase [11]. These enzymes, found on the mucosal surface or within intracellular compartments, can hinder drug permeation through the buccal epithelium, with the extent of interaction being influenced by the specific transport mechanism [12]. Absorption primarily occurs through two pathways, with saliva pH playing a crucial role in influencing drug ionization. Transcellular diffusion, the more common transport mechanism, is particularly affected by the drug’s lipid solubility, favoring drugs in their non-ionized, lipophilic form. On the other hand, paracellular diffusion is preferred for hydrophilic or ionized molecules [8]. Moreover, the residence time of drug formulations in the buccal area, which varies among formulations and patients, plays a crucial role in drug absorption. Many dosage forms face challenges in maintaining therapeutic drug levels due to natural mechanisms in the oral cavity, such as saliva wash and mechanical stress. These mechanisms lead to insufficient exposure time and unpredictable distribution [13]. As such, to achieve therapeutic effects at the target site, it is essential to prolong contact time with the mucosa. This extension of buccal residence time will also reduce the severity of undesirable side effects [12]. To delve deeper into the factors impacting drug absorption, it is essential to consider the balance of hydrophilic and lipophilic properties of the drug and its solubility in buccal fluids. Additionally, external factors like smoking can further complicate buccal absorption [8].

Therefore, for the development of a buccal formulation, a drug candidate should exhibit distinct physicochemical properties. These properties include favorable lipophilicity (typically a log P value in the range of 1.6–3.3) [14], solubility at physiological pH, high potency [15], and ionization characteristics determined by the drug’s pKa and the pH of the environment, with only the non-ionized species capable of effective cell membrane permeation [16] (Table 1). However, the number of suitable candidates for buccal formulations is limited by low drug loading, including molecular size and dose. Generally, smaller molecules facilitate easier transportation across epithelial cell layers, with an ideal molecular weight not exceeding 800 Da [17]. Given the limited volume and surface area for absorption in the buccal region, drug loading is relatively small, with doses typically ranging from 1 to 10 mg, although higher doses may be achievable with optimized formulations. Hence, a suitable drug candidate must possess high potency without causing local irritation [18].

After exploring the distinctive characteristics of drugs for buccal administration and recognizing both the challenges and advantages associated with this delivery method, the focus shifts to the critical role of formulation design and composition in optimizing drug delivery through the buccal route. Buccal delivery, with its potential for increased bioavailability and reduced systemic side effects, necessitates a carefully devised formulation approach. The formulation acts as the guiding force, shaping the release profile, ensuring stability, and determining the overall efficacy of the administered drug. Furthermore, the dynamic nature of the oral mucosa introduces an additional layer of complexity, requiring formulations that adapt to physiological conditions, especially when aiming to enhance the buccal absorption of BCS Class III drugs.

The oral cavity primarily relies on absorption through the non-keratinized sublingual and buccal mucosa, whereas the keratinized palatal and gingival mucosa exhibit lower permeability [20]. The oral mucosal membrane differs from the epithelial membranes in the intestine due to the absence of tight junctions found in most tissues. Instead, the oral mucosa consists of intercellular spaces filled with lipids derived from membrane-coating granules, forming lamellae that serve as the primary barrier against molecular diffusion [21].

To address the challenge posed by the high solubility yet low permeability characteristics of BCS Class III molecules, various formulation approaches have been explored, including the use of mucoadhesive polymers and permeation enhancers [22]. These approaches aim to enhance the interaction between the drug and the biological membrane, thereby improving drug permeability and reducing drug degradation and clearance [23]. Additionally, a thorough comprehension of the potential mode of action of diverse formulation approaches employed in buccal delivery is essential. Excipients, integral constituents of these formulation approaches, have the capability to influence drug stability, permeability, and overall performance. The interplay between excipients and biological barriers, specifically the buccal mucosa, plays a pivotal role in determining drug absorption outcomes. To enhance permeability through the buccal routes, a common approach involves increasing the lipid fluidity of intercellular lipids, thereby enhancing paracellular permeability [23]. Nevertheless, each approach exhibits a distinct mechanism of action, which will be elaborated upon.

## 2. Formulation Approaches and Mechanisms

### 2.1. Mucoadhesive Polymers

The concept of “bioadhesion” was initially introduced to describe the binding of a natural or synthetic macromolecule to epithelial or mucus surfaces. This definition still applies, encompassing the adherence of polymeric materials to biological surfaces (bioadhesives) or mucosal tissue (mucoadhesives) [24]. Mucins, which are highly glucosylated glycoproteins, are the primary component of the mucus layer that covers mucosal surfaces, and they carry negatively charged terminated O-linked oligosaccharide side chains at physiological pH [7].

Successful buccal drug delivery requires the use of mucoadhesive polymers that can adhere to the buccal mucosa and extend the drug’s residence time in the oral cavity. Different classes of polymers, such as cellulose derivatives, high-molecular-weight poly(acrylic acid), and chitosan, as well as protein mucoadhesives, have been investigated for their potential as mucoadhesive polymers in buccal drug delivery [25].

Shivanand et al. [26] conducted a study to enhance the bioavailability of sumatriptan succinate, a BCS Class III drug used to treat migraines. They designed mucoadhesive bilayered buccal tablets using Carbopol 934, Hydroxypropyl methylcellulose (HPMC) K4M, and HPMC K15M mucoadhesive polymers, with an ethyl cellulose backing layer to improve its low oral bioavailability of 15%. The optimized formulation containing Carbopol and HPMC K4M in a 1:1 ratio showed the highest in vitro drug release within 6 h and exhibited 140.78% relative in vivo bioavailability in rabbits, potentially due to reduced first-pass metabolism when administered via the buccal route [26]. In another study by Adhikari et al. [27], hydrophilic polymers were utilized to enhance drug release through the buccal route. This study aimed at formulating sustained-release buccal patches of atenolol using a combination of mucoadhesive hydrophilic polymers such as sodium alginate (SA), HPMC, sodium carboxymethyl cellulose (NaCMC), and Carbopol 934P. The study focused on the impact of different proportions and combinations of these polymers on the physicomechanical properties, mucoadhesive characteristics, in vitro drug release, and ex vivo drug permeation of the formulated patches. The results of the study demonstrated that the atenolol buccal patches exhibited satisfactory physicomechanical and mucoadhesive properties, and the release of the drug was dependent on the proportion of the hydrophilic polymers used. Specifically, the permeation parameters of atenolol through goat buccal mucosa from a buccal patch formulation containing a more hydrophilic polymeric matrix showed the highest permeation flux (30.83 ± 1.23 µg/cm^2^/h) for atenolol [27]. This aligns with Shivanand et al.’s findings, in which hydrophilic polymers not only facilitated mucoadhesion but also contributed to improved drug permeation through the buccal mucosa. Both studies collectively showed that mucoadhesive polymers play a crucial role in extending the residence time of the dosage form in the buccal cavity. This extension is achieved through the hydrophilic properties of the polymer without directly affecting the drug’s chemical properties.

**Figure 1:**
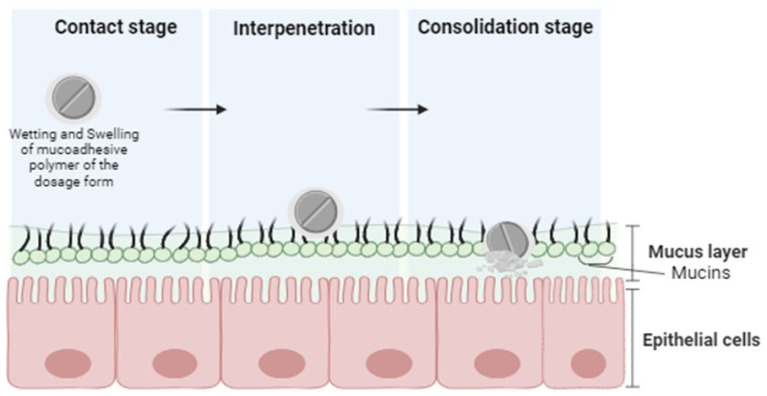
Mechanism of mucoadhesion.

Although the precise mechanisms of polymer attachment to mucosal surfaces are not yet fully understood, physical entanglement and/or chemical interactions, such as electrostatic, hydrophobic, hydrogen bonding, and van der Waals’ interactions, have been proposed as possible theories [28]. These interactions play a crucial role in the formation of a mucoadhesive joint, a bond between a mucoadhesive material and a mucous membrane, occurring in three sequential steps: the contact stage, interpenetration stage, and consolidation stage [9] (Figure 1).

The contact stage occurs when the mucoadhesive and mucosal epithelium come into intimate contact, facilitated by factors such as placing or holding a mucoadhesive material in direct contact with the mucosal surface or adsorbing the mucoadhesive onto the gastrointestinal tract [25]. The physical state and hydration of the materials play a crucial role in this step. The interpenetration stage involves the diffusion of polymer chains into the mucus layer, leading to chain entanglements [29]. Finally, in the consolidation stage, mechanical and chemical interactions contribute to the strengthening of the mucoadhesive joint. Mechanical bonds involve the physical entanglement of polymer chains into the mucus layer, while chemical bonds include hydrogen bonds, electrostatic interactions, covalent bonds, etc. [28]. The mucoadhesive joint is reversible, with failure typically occurring at the interface between the adhesive and mucus layers. Water transport from the tissue to the adhesive affects the residence time, both weakening the adhesive through dilution and increasing mucus cohesion through dehydration [30].

Multiple mucoadhesive excipients have been investigated, each utilizing distinct mechanisms to enhance absorption. One example is polycarbophil, a water-soluble polymer that contains hydroxyl groups capable of forming hydrogen bonds with mucin glycoproteins. These hydrogen bonds provide adhesion to the mucosal membrane, thereby prolonging the contact time and facilitating enhanced absorption [31]. In a study by Wasnik et al. [32], the application of polycarbophil and thiolated polycarbophil as mucoadhesive polymer buccal tablets was investigated. Using selegiline hydrochloride, a BCS I/III drug prescribed for individuals with Parkinson’s disease, as a model drug, the study aimed to bypass first-pass metabolism and enhance the bioavailability of the drug. The results demonstrated that buccal tablets formulated with thiolated polycarbophil (PCP-cys) exhibited improved mucoadhesion and controlled drug release compared to those formulated with polycarbophil. Permeation data indicated significantly higher apparent permeability values for matrices incorporating the PCP-cys polymer, emphasizing the permeation-enhancing properties conferred by thiolation. This enhancement is attributed to the intimate contact between the thiolated polymer and the mucosal surface, providing a comprehensive understanding of the impact of mucoadhesive excipients on drug delivery [32].

Another example are carbomers, such as carbomer 934 and carbomer 974P, widely employed mucoadhesive excipients known for their excellent mucoadhesive properties. They form a gel-like matrix upon hydration, which adheres to the mucosal membrane through hydrogen bonding with mucin glycoproteins [33]. Chitosan, a natural mucoadhesive polymer derived from chitin, exhibits cationic properties that enable electrostatic interactions with negatively charged mucin glycoproteins [34]. This electrostatic attraction enhances mucoadhesion and subsequently improves drug absorption. Another example is sodium alginate, a biopolymer derived from seaweed, which forms a viscous gel upon contact with calcium ions. This gel-like structure promotes mucoadhesion, thereby facilitating prolonged contact between the drug formulation and the mucosal membrane, leading to enhanced absorption [35].

### 2.2. Permeation Enhancers

Permeation enhancers are excipients that modify the physicochemical properties of the barrier. In the context of drug absorption through the buccal route, the partition coefficient emerges as a critical factor. Drugs that meet various parameters for suitable delivery through the buccal route except for the desired partition coefficient require additional support to enhance penetration. This is where the co-administration of penetration enhancers with the drug becomes crucial, as it is employed to enhance drug penetration into the buccal mucosa. Notably, the action of penetration enhancers varies based on their chemical nature, and their effectiveness is specific to different drugs and mucosal surfaces [36].

Due to the significant barrier presented by the buccal epithelium, permeation enhancers are proposed to reduce drug degradation and clearance, promote stronger drug-membrane interactions, and improve drug permeability by augmenting drug absorption through various mechanisms [23]. These mechanisms include modifying mucus rheology, increasing membrane fluidity, overcoming enzymatic barriers, or increasing the thermodynamic activity of the drug [22]. The combination of saliva and the viscoelastic properties of mucus can create a barrier to drug absorption. To address this, enhancers are used to modify mucus rheology by reducing the viscosity of both mucus and saliva. This modification facilitates drug permeation through the mucosal barrier, enhancing absorption. However, it is important to note that while reducing the viscosity of saliva may enhance drug permeation by decreasing the mucus barrier, it could also reduce the residence time of the buccal film due to the enhanced washing effect of saliva. This trade-off must be carefully considered in the formulation design to balance the benefits of enhanced absorption with the potential disadvantage of shortened residence time. Another strategy employed by enhancers involves altering the fluidity of the lipid bilayer membrane. This disruption increases membrane fluidity, promoting drug absorption. Enzymatic barriers within the buccal mucosa can also restrict drug permeation, but most permeation enhancers inhibit the activity of peptidases and proteases present in the buccal mucosa, effectively overcoming this enzymatic barrier. Additionally, enhancers can alter the partition coefficient of drugs; increasing their solubility leads to a higher thermodynamic activity of the drugs, ultimately improving their absorption [22,37].

Surfactants, bile salts, cyclodextrins, and fatty acids have been studied as permeation enhancers in the development of buccal dosage forms, with observed permeability increases for both small drug molecules and biologics in various bioassays [38]. It is worth noting that only a limited number of permeation enhancers that have a track record of safe use in humans have advanced to clinical testing [39].

The buccal delivery of insulin, much like other proteins and peptides, faces challenges in achieving adequate bioavailability due to issues such as metabolic breakdown or limited membrane permeability. To address this, Sahni et al. [40] aimed to enhance insulin bioavailability by creating a controlled-release buccoadhesive patch using solvent casting. This patch utilized sodium carboxymethylcellulose-DVP as a bioadhesive and controlled release matrix-forming polymer. The study explored various permeability enhancers, such as β-cyclodextrin, the surfactant sodium lauryl sulphate, the bile salts sodium glycocholate and sodium deoxycholate, and the fatty acid glyceryl monolaurate. Notably, the study found that a 5% (*w*/*v*) concentration of the bile salt sodium deoxycholate was the most effective enhancer, increasing insulin permeation from 6.63% to 10.38% over 6 h [40].

It is crucial to understand the role of bile salts in drugs, as their influence varies depending on factors such as the drug’s characteristics and the interaction between micelles and the physiological environment [41]. Bile salts play a crucial role in enhancing drug movement through different routes. They facilitate the transport of hydrophobic compounds through the transcellular route and enhance the movement of hydrophilic drugs, such as insulin, through the paracellular route [42]. This is achieved by incorporating bile acids into cell membranes, creating hydrophilic pores that allow water passage. Furthermore, bile salts can improve drug penetration through paracellular pathways by binding to calcium ions, leading to the opening of intercellular spaces. They also have a mucolytic effect, breaking down mucus barriers and contributing to improved drug absorption [41]. However, it is important to note that the mucolytic effect may also weaken the bonds between mucin and mucoadhesive polymers, potentially reducing the residence time of the formulation. This effect should be carefully considered in the formulation design to balance the benefits of enhanced absorption with the potential disadvantage of shortened residence time.

Expanding on this, the discussion transitions to the permeation-enhancing efficacy of cationic and anionic surfactants compared to non-ionic compounds. Despite their greater efficacy, these surfactants come with higher levels of toxicity [43]. Their mechanisms include protein denaturation, tissue swelling, and lipid extraction [44]. For example, sodium lauryl sulphate interacts with the lipid bilayer of membranes, disrupting the packing of lipid molecules and increasing the fluidity of the bilayer, facilitating drug diffusion through the membrane [22]. The concentration of surfactants significantly influences drug permeation, with lower concentrations below the critical micellar concentration (CMC) improving drug penetration, while higher concentrations above the CMC may reduce absorption by causing drug entrapment in micelles [45]. This dual exploration of bile salts and surfactants highlights the complexity and importance of understanding these mechanisms in optimizing drug delivery systems.

Another study, conducted by Prasanth et al. [46], aimed to assess the impact of various permeation enhancers, specifically fatty acids like linoleic acid (LA), isopropyl myristate (IPM), and oleic acid (OA), on the buccal absorption of salbutamol sulphate (SS) from buccal patches. The objective was to augment the bioavailability and therapeutic effect of SS by avoiding first-pass metabolism in the liver and gut wall. Buccal patches composed of different polymer combinations, such as HPMC, carbopol, polyvinyl alcohol (PVA), polyvinyl pyrollidone (PVP), sodium carboxymethyl cellulose (NaCMC), acid and water-soluble chitosan (CHAS and CHWS), and Eudragit-L100 (EU-L100), were tested. The results showed that OA was the most effective permeation enhancer, increasing the flux by more than 8 fold compared to patches without enhancers in HPMC-based buccal patches when PEG-400 was used as the plasticizer [46]. Expanding upon the role of fatty acids, particularly in buccal delivery, their impact on drug absorption is influenced by factors such as the presence and position of double bonds, isomer type (cis or trans), and degree of branching [47]. Despite ongoing research, the underlying mechanism of fatty acids in buccal delivery has not yet been definitively established. But it is hypothesized that their presence within a membrane could potentially alter the interactions between hydrocarbon chains, such as van der Waals forces. Additionally, it is theorized that fatty acids may facilitate bonding with neighboring moieties through their carboxyl groups [45].

In conjunction with the investigation into fatty acids, the role of cyclodextrins as promising penetration enhancers for the buccal route is highlighted by their capacity to modify the physical, chemical, and biological properties of drug molecules through the formation of inclusion complexes [48,49]. This potential was demonstrated in a study by Yoo et al. [50], which investigated the absorption and distribution of the BCS III drug clomipramine [51] in rats using various administration routes, including sublingual and oral methods. The study revealed that sublingual administration resulted in significantly higher bioavailability (36.2%) compared to oral dosing (24.8%) in conscious rats. To enhance the sublingual bioavailability of clomipramine, a formulation incorporating the permeation enhancer 2-hydroxypropyl β-cyclodextrin was developed. Following administration of this sublingual formulation in rats, both C_max_ and AUC were substantially increased by 3.3 and 1.6 fold, respectively, on a dose-normalized basis compared to formulations without the permeation enhancer. The introduction of the permeation enhancer significantly elevated the bioavailability of clomipramine to 57.1%, representing a >192% increase over oral administration [50]. These findings highlight the potential of cyclodextrins as an effective strategy for enhancing the bioavailability of BCS III drugs, exemplified here by clomipramine.

While the precise mechanism for the observed enhancement was not specifically addressed in the current study, previous research has demonstrated that β-cyclodextrin derivatives can augment transmucosal drug absorption by transiently altering membrane permeability and overcoming the diffusion barrier. Moreover, cyclodextrins, as a class of penetration enhancers, have been found to enhance drug permeation by increasing drug availability and stability at the biological barrier’s surface [49]. Specifically, these penetration enhancers can permeate the buccal mucosa, forming inclusion complexes with hydrophobic lipids from the cellular membrane [52]. Through interactions with these lipids, cyclodextrins can modify buccal mucosa permeability, offering a comprehensive understanding of their potential in improving drug delivery systems.

### 2.3. Prodrug

Prodrugs are modified versions of active drugs that can convert into their active form within the human body. This allows them to overcome various obstacles, such as biopharmaceutical, pharmacokinetic, or pharmacodynamic issues. The use of prodrugs is becoming increasingly popular for achieving optimal oral bioavailability and therapeutic effects, with approximately 10% of all commercially available medicines considered prodrugs [53]. However, to produce the desired therapeutic effects, prodrugs must be activated to create the active parent drug. This activation can be nonspecific, but understanding the potential enzymes involved in the process is crucial for designing prodrugs that are both selective and effective. The prodrug approach can be used to optimize newly discovered chemical entities as well as to improve the properties of existing drugs [54]. Traditional prodrug design utilizes covalent binding of the parent drug to either hydrophilic or lipophilic functional groups to enhance the drug’s solubility and permeability, leading to improved properties and overcoming challenges within the body [55]. Although lacking specificity, the traditional approach is effective in altering drug pharmacokinetics, achieving prolonged drug release, and mitigating toxicity [53,56]. On the other hand, the modern approach to prodrug design considers molecular and cellular parameters, such as membrane transporters and protein expression, to target specific molecules in the body [57]. A carrier is covalently attached to the parent drug to selectively target enzymes or transporters. This approach can regulate the release of the parent drug from the prodrug, along with the potential to direct the active drug to its intended sites [53]. Irrespective of the method employed, prodrugs must undergo an activation process to transform into the active parent drug and produce their intended pharmacological effects. This activation can occur through chemical or enzyme-mediated conversion.

For example, the bioavailability of morphine via buccal administration is limited, owing to its poor lipophilicity and polar properties at physiological pH. To address this issue, Christrup et al. [58] explored a prodrug approach aimed at enhancing the penetration of morphine through the buccal mucosa by esterifying its 3-phenolic group. In their study, enzymatic hydrolysis occurred in all morphine esters tested, but the acetyl prodrug (morphine-3 acetate) was observed to be the most stable derivative in both plasma and saliva. Interestingly, the ester prodrugs demonstrated permeability through porcine buccal mucosa, while the parent drug was not detected to any measurable extent. Notably, the use of ester prodrugs with higher lipophilicity resulted in a considerable improvement in the permeation of morphine. Their findings suggest that esterification could be a promising approach to enhance the bioavailability of morphine via buccal administration [58]. In a parallel context, the antiviral drug Acyclovir, categorized as a BCS III drug, encounters challenges in oral administration due to its low bioavailability of only 20%, resulting in moderate antiviral efficacy [59]. To address this, the valine ester prodrug valacyclovir was developed, strategically targeting the intestinal oligopeptide transporter 1 (PepT1). Valacyclovir’s efficacy relies on the rapid conversion to acyclovir within the body. This prodrug remains stable in the gastrointestinal tract, allowing improved absorption through PEPT1. It undergoes enzymatic activation within cells, and this conversion is facilitated by a specific enzyme called human valacyclovirase, which belongs to the serine hydrolase family [53]. These examples illustrate the diverse applications of prodrug strategies in overcoming bioavailability challenges for different drugs.

### 2.4. Ion Pairing

The ion-pairing approach, involving a highly charged, polar molecule combined with a lipophilic counterion, forms an ion pair capable of passively permeating cell membranes [60,61]. This approach bypasses the need for prodrug uptake via transporters and activation through specific enzymes [62]. Expanding on this concept, the ion-pairing approach combines the molecule of interest with oppositely charged ions to form a neutral ion pair. A recommended pKa difference of up to 3 units allows for sufficient ionization, producing counterions that form non-ionized complexes and enhance permeability without altering the structure and function of the drug [63]. Ion pairs act as a single unit and easily integrate into the membrane, increasing the molecule’s lipophilicity, membrane permeability, and absorption [64]. This enhanced permeability is achieved by forming a complex held together by Coulombic attractive forces rather than covalent bonding [65]. The strategy involves co-administering an excess of a counterion, and after absorption into the bloodstream, the ion pair dissociates upon dilution or displacement, simplifying drug delivery by eliminating the need for transporter uptake and enzyme activation [61]. To ensure effectiveness, a high association constant (K) is crucial, neutralizing the charge on drug molecules and allowing passive diffusion of the ion-pair complex across biological membranes [60].

The utilization of counterions plays a pivotal role in enhancing the permeability of various class drugs. An exemplary instance is the frequently employed counterion, 1-hydroxy-2-naphthoic acid (HNAP), known for its high lipophilicity and robust binding constants, counterbalancing the inherent polarity of several BCS Class III drugs [66], thereby improving their solubility and permeability. Miller et al. [64] provided compelling evidence supporting this relationship, demonstrating that the addition of HNAP as a counterion correlated with an increase in membrane permeability for the BCS Class III drug, phenformin. The researchers observed that as the amount of HNAP counterion increased, the lipophilicity of the drug also showed a corresponding rise. This correlation between enhanced permeability and increased drug lipophilicity highlights the impact of the attached counterion on improving the membrane permeability of phenformin [64]. Similarly, Bashyal et al. [67] conducted a study where sodium glycodeoxycholate (SGDC), an anionic bile salt, was employed as a counterion for the formation of hydrophobic ion-pairing nanocomplexes with insulin as the model peptide. These nanocomplexes were created through a combination of electrostatic and hydrophobic interactions. The results indicated that the nanocomplex with a higher concentration of SGDC significantly enhanced the transport of insulin across TR146 cell layers and porcine buccal tissues. Compared to insulin solution without the counterion, the nanocomplex exhibited a remarkable 3.00-fold and 51.76-fold increase in permeability coefficient for TR146 cell layers and porcine buccal tissue, respectively [67]. This highlights the crucial role of counterions, such as HNAP and SGDC, in modulating drug properties and improving their transport across biological barriers.

The effective formation of ion-pair complexes, as elucidated by the proton-transfer model proposed by Huyskens and Zeegers-Huyskens, relies on key factors. To ensure favorable outcomes, a minimum ΔpKa of ≥2.5 between the base and acid is crucial, with optimal pairing more likely when employing strong functional groups like phosphate or sulphate (with low pKa values) and quaternary amine or guanidinium groups (with high pKa values). However, the unique nature of ionizable groups in peptide drugs challenges the use of individual residue pKa values as a parameter. Instead, the isoelectric point (pI) of peptides proves to be a more appropriate determinant in this context [68].

This perspective on peptide characteristics becomes particularly relevant when considering pH-dependent ion-pair formation, as highlighted in a study by Iyire et al. [69]. The investigation focused on insulin, possessing a pI of 5.35. At pH 7.4, approximately two units above insulin’s pI, the peptide manifests a net negative charge. This charge state facilitates the formation of ion pairs with basic amino acids like arginine, lysine, and histidine, which are positively charged below their respective pKa values. Interestingly, the study also noted a similar phenomenon with acidic amino acids (aspartic and glutamic acid) at low concentrations, attributed to the presence of the protonated alpha amino group. However, the concentration-dependent decrease in pH, resulting from high concentrations of acidic amino acids, proved to impact the overall charge of the peptide, subsequently influencing the formation of the ion-pair complex [69]. This intricate interplay between peptide characteristics and pH dynamics emphasizes the importance of understanding the nuanced factors governing ion-pair complex formation in peptide drugs.

### 2.5. pH Modifiers

Modifying the microenvironmental pH has become a widely utilized technique to enhance the dissolution of BCS III drugs. This technique involves the incorporation of pH modifiers into formulations to establish an ideal pH level within and around the solid dosage forms. Notably, the physiological environment of the oral cavity is unique compared to the gastrointestinal (GI) tract with respect to drug dissolution and permeation. Specifically, human saliva possesses a pH range of 6.2 to 7.6, which creates a neutral pH environment suitable for establishing the desired microenvironmental pH [15]. Furthermore, the slow release of pH modifiers from formulations due to the limited volume (an average of 0.8 to 1.1 mL) and low secretion rate (0.35 to 2.00 mL/min) of human saliva maintains the ideal microenvironmental pH [15]. As such, microenvironmental pH modifications can be effectively implemented in buccal dosage forms to improve the absorption of BCS III drugs by impacting solubility and permeability [15]. When buccal formulations adhere to the oral mucosa, a narrow space is formed between the formulation and the mucous membrane, which is crucial for effective drug absorption as the drug must be released, dissolve within this space, and subsequently permeate through the mucosal membrane. They can be included in the formulation to adjust the pH within the formulation itself and in the space, impacting drug release by altering drug solubility, solubility in the space, and permeation across the mucosa through their influence on drug dissociation [15,70]. There are two primary techniques that are frequently employed to modify the pH of buccal formulations: either by the incorporation of acidifying or alkalizing agents or the use of buffer agents [15].

The incorporation of acidifying or alkalizing agents directly into the formulation modifies the pH in the microenvironment where the drug is released, positively impacting its solubility and absorption. This pH adjustment enhances drug release and absorption by facilitating pH-dependent solubility. Equally, the use of alkalizing agents can create an alkaline microenvironment, potentially improving drug permeation through the oral mucosa. Buffering agents play a crucial role in maintaining a stable pH, forming a buffer system that resists pH changes caused by the release of acidic or basic components from the formulation. By preventing significant pH fluctuations, buffering agents ensure optimal conditions for efficient drug absorption.

In a study by Qalaji et al. [71], fast-disintegrating sublingual tablets of the weak base drug atropine sulphate (AS) were tested as a potential alternative dosage form for the treatment of organophosphate toxicity. The primary objective of the study was to establish a pH permeability profile for AS, with the aim of identifying the optimal pH for sublingual AS permeability and the ideal alkalizer to include in AS formulations to achieve this pH. Increasing the pH from the average saliva pH of 6.8 to 8 through the use of an alkalizer significantly enhanced AS permeability by reducing ionization and increasing lipophilicity. Notably, the incorporation of a transcellular enhancer or an alkalizer resulted in a substantial enhancement of AS permeability, with the combination of 2% sodium bicarbonate and 1% sodium dodecyl sulphate leading to the most significant improvement (up to twelve fold). The study concluded that the inclusion of an alkalizing excipient modified sublingual AS permeability by adjusting the pH of the absorption microenvironment to an optimal range for AS absorption. This, in turn, reduced the absorption variability influenced by individual variations in oral pH [71].

## 3. Expert Opinion

Buccal drug delivery stands as a promising avenue for enhancing the bioavailability and therapeutic efficacy of poorly permeable drugs, particularly those falling within BCS Class III. The buccal mucosa facilitates systemic drug absorption through the external jugular vein, bypassing the first-pass effect, and offers advantages such as easy accessibility, robust vascularity, relative permeability, and low enzymatic activity compared to other mucosal membranes [6]. However, challenges inherent in the anatomical features, physiology, and hydrodynamics of the buccal cavity impede the seamless delivery of these drugs.

**Figure 2:**
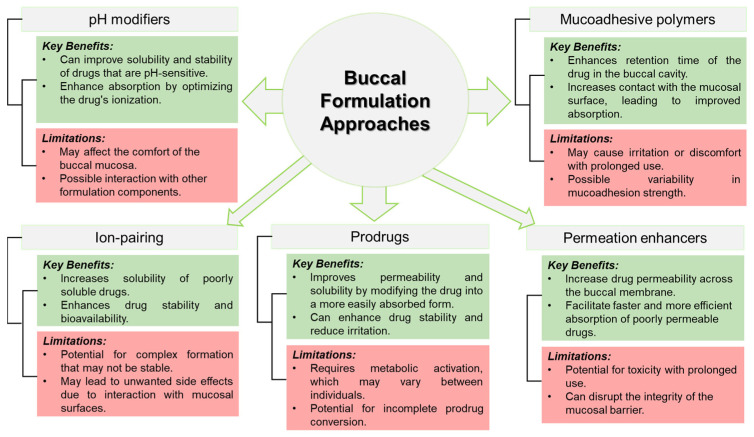
Summary depicting the key advantages and limitations for each strategy.

Current published research aims to overcome constraints in buccal drug delivery through innovative formulations, paving the way for significant advancements in the use of BCS III drugs in buccal administration (Figure 2). Exploring the application of mucoadhesive polymers, permeation enhancers, ion pairing, prodrugs, and pH modifiers reveals diverse potential solutions to enhance drug absorption (Table 2). Nevertheless, the translation of these strategies into clinical success demands a crucial emphasis on a patient-centric approach that customizes formulations to meet specific therapeutic needs. While navigating the complexities of formulation design, it is essential to maintain a continuous focus on safety considerations, keeping them at the forefront of the process.

Extensive research efforts have been directed toward addressing the unique needs of diverse patient populations with various medical conditions, ranging from migraines to mental illnesses. This has led to notable solutions, including the incorporation of formulation approaches into traditional dosage forms such as buccal tablets or films. Such integration facilitates a significant improvement in patient outcomes compared to traditional solid dosage forms, especially given the challenges the latter encounter with saliva and swallowing. These challenges impact consistent mucosal contact, resulting in unpredictable drug absorption. The layered structure of the buccal epithelium further complicates the situation, highlighting the importance of these advancements in everyday healthcare.

The market currently offers some buccal patches, including those approved by the FDA, but there remains a limited number of innovative formulations reaching advanced clinical development stages. However, the innovation that focuses on the permeation enhancer approach has demonstrated its effectiveness in improving the absorption of the BCS Class III drug peptide Octreotide within the small intestine. This has led to the emergence of Mycapssa^®^, an FDA-approved oral peptide, as a compelling alternative to the discomfort-inducing subcutaneous injectable formulation in 2020. This oral formulation surpasses the limitations typically associated with injectable products, offering a more patient-friendly solution. By utilizing the permeation enhancer sodium caprylate, permeation is enhanced through its temporary and reversible mechanism. This process induces the reorganization of tight junction proteins, including Claudin, and is thought to operate via paracellular modulation within the tight junction structure [72].

As the field of buccal drug delivery continues to evolve, the synergistic integration of conventional dosage forms with formulation approaches represents a potential strategy to improve the limited permeability and therapeutic effectiveness of BCS Class III drugs through the buccal mucosa. By addressing the inherent challenges through a holistic and patient-centered lens, the prospects for successfully delivering BCS III drugs via the buccal route are propelled toward transformative breakthroughs in drug development and therapeutic outcomes.

## 4. Conclusions

In conclusion, this review discusses various formulation approaches for enhancing buccal drug delivery of BCS III compounds, with a focus on their potential to improve absorption. The mechanistic roles of the formulation approaches are examined, providing valuable insights into permeability enhancement in the buccal region. This comprehensive examination highlights the potential of merging traditional drug forms with innovative strategies to enhance buccal delivery for BCS III compounds, offering a promising resolution to absorption challenges.

## Figures and Tables

**Table 1:** Ideal drug characteristics for optimal pre-gastric drug absorption [19].

Ideal Characteristics for Pre-Gastric Absorption
Less than 20 mg dose
Small-to-moderate molecular weight (<800 Da)
Soluble in water/saliva
Partially non-ionized at pH 6.8 (oral cavity pH)
Ability to diffuse and partition into the upper GI tract

**Table 2:** Summary of strategies to improve buccal permeability.

Formulation Approach	Key Findings	Effect on Permeation	Mechanism of Action
Mucoadhesive Polymers	Enhances residence time of the drug on the mucosal surface, allowing for prolonged contact and increased absorption.	Potential to increase permeation due to extended exposure at the absorption site.	Binds to mucosal surface, reducing washout and sustaining release.
Permeation Enhancers	Increases membrane permeability through surfactants or fatty acids, improving drug transport.	Enhances permeability by masking the drug’s charge.	Forms ion pairs that pass through membranes with reduced repulsion.
Prodrugs	Enhances drug solubility, stability, and targeted absorption.	Facilitates drug absorption by releasing active moiety at target site.	Converts inactive prodrug to active form at absorption site.
Ion pairing	Forms a neutral ion pair, increasing lipophilicity and membrane permeability.	Can increase permeation by altering membrane structure or reducing mucus viscosity.	Alters cell membrane properties or reduces mucus viscosity.
pH modifiers	Adjusts the microenvironmental pH to enhance drug solubility and absorption, particularly effective for BCS III drugs in buccal formulations by creating an ideal pH environment within and around the solid dosage forms.	Enhances permeability and solubility through pH-dependent dissolution and absorption.	Uses acidifying or alkalizing agents or buffering systems to maintain optimal pH levels, facilitating drug release and permeability.

## References

[1] Sahoo D., Bandaru R., Samal S.K., Naik R., Kumar P., Kesharwani P., Dandela R., Kesharwani P., Taurin S., Greish K. (2020). Oral Drug Delivery of Nanomedicine. Theory and Applications of Nonparenteral Nanomedicines.

[2] Bocci G., Oprea T.I., Benet L.Z. (2022). State of the Art and Uses for the Biopharmaceutics Drug Disposition Classification System (BDDCS): New Additions, Revisions, and Citation References. AAPS J..

[3] Alqahtani M.S., Kazi M., Alsenaidy M.A., Ahmad M.Z. (2021). Advances in Oral Drug Delivery. Front. Pharmacol..

[4] Asad M., Rasul A., Abbas G., Shah M.A., Nazir I. (2023). Self-Emulsifying Drug Delivery Systems: A Versatile Approach to Enhance the Oral Delivery of BCS Class III Drug via Hydrophobic Ion Pairing. PLoS ONE.

[5] Papich M.G., Martinez M.N. (2015). Applying Biopharmaceutical Classification System (BCS) Criteria to Predict Oral Absorption of Drugs in Dogs: Challenges and Pitfalls. AAPS J..

[6] Sudhakar Y., Kuotsu K., Bandyopadhyay A.K. (2006). Buccal Bioadhesive Drug Delivery—A Promising Option for Orally Less Efficient Drugs. J. Control. Release.

[7] Gandhi R.B., Robinson J.R. (1994). Oral Cavity as a Site for Bioadhesive Drug Delivery. Adv. Drug Deliv. Rev..

[8] Hua S. (2019). Advances in Nanoparticulate Drug Delivery Approaches for Sublingual and Buccal Administration. Front. Pharmacol..

[9] Zhang H., Zhang J., Streisand J.B. (2002). Oral Mucosal Drug Delivery: Clinical Pharmacokinetics and Therapeutic Applications. Clin. Pharmacokinet..

[10] Pickering G., Macian N., Libert F., Cardot J.M., Coissard S., Perovitch P., Maury M., Dubray C. (2014). Buccal Acetaminophen Provides Fast Analgesia: Two Randomized Clinical Trials in Healthy Volunteers. Drug Des. Devel Ther..

[11] Garren K.W., Repta A.J. (1988). Buccal Drug Absorption. I. Comparative Levels of Esterase and Peptidase Activities in Rat and Hamster Buccal and Intestinal Homogenates. Int. J. Pharm..

[12] Jacob S., Nair A.B., Boddu S.H.S., Gorain B., Sreeharsha N., Shah J. (2021). An Updated Overview of the Emerging Role of Patch and Film-Based Buccal Delivery Systems. Pharmaceutics.

[13] Rossi S., Sandri G., Caramella C.M. (2005). Buccal Drug Delivery: A Challenge Already Won?. Drug Discov. Today Technol..

[14] Smart J.D. (2005). Buccal Drug Delivery. Expert. Opin. Drug Deliv..

[15] He S., Mu H. (2023). Microenvironmental PH Modification in Buccal/Sublingual Dosage Forms for Systemic Drug Delivery. Pharmaceutics.

[16] Lam J.K.W., Cheung C.C.K., Chow M.Y.T., Harrop E., Lapwood S., Barclay S.I.G., Wong I.C.K. (2020). Transmucosal Drug Administration as an Alternative Route in Palliative and End-of-Life Care during the COVID-19 Pandemic. Adv. Drug Deliv. Rev..

[17] Lam J.K.W., Xu Y., Worsley A., Wong I.C.K. (2014). Oral Transmucosal Drug Delivery for Pediatric Use. Adv. Drug Deliv. Rev..

[18] Gilhotra R.M., Ikram M., Srivastava S., Gilhotra N. (2014). A Clinical Perspective on Mucoadhesive Buccal Drug Delivery Systems. J. Biomed. Res..

[19] Bala R., Pawar P., Khanna S., Arora S. (2013). Orally Dissolving Strips: A New Approach to Oral Drug Delivery System. Int. J. Pharm. Investig..

[20] Squier C.A. (1991). The Permeability of Oral Mucosa. Crit. Rev. Oral Biol. Med..

[21] Wertz P.W. (2021). Roles of Lipids in the Permeability Barriers of Skin and Oral Mucosa. Int. J. Mol. Sci..

[22] Chinna Reddy P., Chaitanya K.S.C., Madhusudan Rao Y. (2011). A Review on Bioadhesive Buccal Drug Delivery Systems: Current Status of Formulation and Evaluation Methods. DARU J. Pharm. Sci..

[23] Veuillez F., Kalia Y.N., Jacques Y., Deshusses J., Buri P. (2001). Factors and Strategies for Improving Buccal Absorption of Peptides. Eur. J. Pharm. Biopharm..

[24] Salamat-Miller N., Chittchang M., Johnston T.P. (2005). The Use of Mucoadhesive Polymers in Buccal Drug Delivery. Adv. Drug Deliv. Rev..

[25] Khutoryanskiy V.V. (2011). Advances in Mucoadhesion and Mucoadhesive Polymers. Macromol. Biosci..

[26] Shivanand K., Sa R., Nizamuddin S., Jayakar B. (2011). In Vivo Bioavailability Studies of Sumatriptan Succinate Buccal Tablets. DARU J. Pharm. Sci..

[27] Adhikari S.N.R., Nayak B.S., Nayak A.K., Mohanty B. (2010). Formulation and Evaluation of Buccal Patches for Delivery of Atenolol. AAPS PharmSciTech.

[28] Laffleur F. (2014). Mucoadhesive Polymers for Buccal Drug Delivery. Drug Dev. Ind. Pharm..

[29] Shaikh R., Raj Singh T., Garland M., Woolfson A., Donnelly R. (2011). Mucoadhesive Drug Delivery Systems. J. Pharm. Bioallied Sci..

[30] Kavitha K., Rupesh Kumar M., Jagadeesh Singh S. (2011). Novel Mucoadhesive Polymers—A Review. J. Appl. Pharm. Sci..

[31] Zhang Q. (2020). Role of Polymer Physicochemical Properties on In Vitro Mucoadhesion. Ph.D. Thesis.

[32] Wasnik M.N., Godse R.D., Nair H.A. (2014). Development and Evaluation of Buccoadhesive Tablet for Selegiline Hydrochloride Based on Thiolated Polycarbophil. Drug Dev. Ind. Pharm..

[33] Bakhrushina E., Anurova M., Demina N., Kashperko A., Rastopchina O., Bardakov A., Krasnyuk I. (2020). Comparative Study of the Mucoadhesive Properties of Polymers for Pharmaceutical Use. Open Access Maced. J. Med. Sci..

[34] Silva C.A., Nobre T.M., Pavinatto F.J., Oliveira O.N. (2012). Interaction of Chitosan and Mucin in a Biomembrane Model Environment. J. Colloid. Interface Sci..

[35] Chatterjee B., Amalina N., Sengupta P., Mandal U.K. (2017). Mucoadhesive Polymers and Their Mode of Action: A Recent Update. J. Appl. Pharm. Sci..

[36] Patel P.S., Parmar A.M., Doshi N.S., Patel H.V., Patel R.R., Nayee C. (2013). Buccal Drug Delivery System: A Review. Int. J. Drug Dev. Res..

[37] Ibrahim Y.H.E.Y., Regdon G., Hamedelniel E.I., Sovány T. (2020). Review of Recently Used Techniques and Materials to Improve the Efficiency of Orally Administered Proteins/Peptides. DARU J. Pharm. Sci..

[38] Morales J.O., Brayden D.J. (2017). Buccal Delivery of Small Molecules and Biologics: Of Mucoadhesive Polymers, Films, and Nanoparticles. Curr. Opin. Pharmacol..

[39] Fantini A., Giulio L., Delledonne A., Pescina S., Sissa C., Nicoli S., Santi P., Padula C. (2023). Buccal Permeation of Polysaccharide High Molecular Weight Compounds: Effect of Chemical Permeation Enhancers. Pharmaceutics.

[40] Sahni J., Raj S., Ahmad F.J., Khar R.K. (2008). Design and In Vitro Characterization of Buccoadhesive Drug Delivery System of Insulin. Indian J. Pharm. Sci..

[41] Stojančević M., Pavlović N., Goločorbin-Kon S., Mikov M. (2013). Application of Bile Acids in Drug Formulation and Delivery. Front. Life Sci..

[42] Sattar M., Sayed O.M., Lane M.E. (2014). Oral Transmucosal Drug Delivery—Current Status and Future Prospects. Int. J. Pharm..

[43] Som I., Bhatia K., Yasir M. (2012). Status of Surfactants as Penetration Enhancers in Transdermal Drug Delivery. J. Pharm. Bioallied Sci..

[44] Siegel I.A., Gordon H.P. (1986). Surfactant-Induced Alterations of Permeability of Rabbit Oral Mucosa in Vitro. Exp. Mol. Pathol..

[45] Ganem-Quintanar A., Kalia Y.N., Falson-Rieg F., Buri P. (1997). Mechanisms of Oral Permeation Enhancement. Int. J. Pharm..

[46] Prasanth V.V., Puratchikody A., Mathew S.T., Ashok K.B. (2014). Effect of Permeation Enhancers in the Mucoadhesive Buccal Patches of Salbutamol Sulphate for Unidirectional Buccal Drug Delivery. Res. Pharm. Sci..

[47] Sharma S., Kulkarni J., Pawar A.P. (2006). Permeation Enhancers in the Transmucosal Delivery of Macromolecules. Pharmazie.

[48] Sharma N., Baldi A. (2016). Exploring Versatile Applications of Cyclodextrins: An Overview. Drug Deliv..

[49] Masson M., Loftsson T., Masson G., Stefansson E. (1999). Cyclodextrins as Permeation Enhancers: Some Theoretical Evaluations and in Vitro Testing. J. Control. Release.

[50] Yoo S.D., Yoon B.M., Lee H.S., Lee K.C. (1999). Increased Bioavailability of Clomipramine after Sublingual Administration in Rats. J. Pharm. Sci..

[51] Marzo M., Ciccarelli R., Di Iorio P., Giuliani P., Caciagli F., Marzo A. (2016). Synergic Development of Pharmacokinetics and Bioanalytical Methods as Support of Pharmaceutical Research. Int. J. Immunopathol. Pharmacol..

[52] Figueiras A., Pais A.A.C.C., Veiga F.J.B. (2010). A Comprehensive Development Strategy in Buccal Drug Delivery. AAPS PharmSciTech.

[53] Markovic M., Ben-Shabat S., Dahan A. (2020). Prodrugs for Improved Drug Delivery: Lessons Learned from Recently Developed and Marketed Products. Pharmaceutics.

[54] Hussain M.A., Aungst B.J., Koval C.A., Shefter E. (1988). Improved Buccal Delivery of Opioid Analgesics and Antagonists with Bitterless Prodrugs. Pharm.Res. Off. J. Am. Assoc. Pharm. Sci..

[55] Stella V.J., Nti-Addae K.W. (2007). Prodrug Strategies to Overcome Poor Water Solubility. Adv. Drug Deliv. Rev..

[56] Dahan A., Khamis M., Agbaria R., Karaman R. (2012). Targeted Prodrugs in Oral Drug Delivery: The Modern Molecular Biopharmaceutical Approach. Expert. Opin. Drug Deliv..

[57] Dahan A., Zimmermann E.M., Ben-Shabat S. (2014). Modern Prodrug Design for Targeted Oral Drug Delivery. Molecules.

[58] Christrup L.L., Christensen C.B., Friis G.J., Jorgensen A. (1997). Improvement of Buccal Delivery of Morphine Using the Prodrug Approach. Int. J. Pharm..

[59] Zhang Y., Gao Y., Wen X., Ma H. (2014). Current Prodrug Strategies for Improving Oral Absorption of Nucleoside Analogues. Asian J. Pharm. Sci..

[60] Dave V.S., Gupta D., Yu M., Nguyen P., Varghese Gupta S. (2017). Current and Evolving Approaches for Improving the Oral Permeability of BCS Class III or Analogous Molecules. Drug Dev. Ind. Pharm..

[61] Suresh P., Paul S. (2011). Ion-Paired Drug Delivery: An Avenue for Bioavailability Improvement. Sierra Leone J. Biomed. Res..

[62] Samiei N., Shafaati A., Zarghi A., Moghimi H.R., Foroutan S.M. (2014). Enhancement and in Vitro Evaluation of Amifostine Permeation through Artificial Membrane (PAMPA) via Ion Pairing Approach and Mechanistic Selection of Its Optimal Counter Ion. Eur. J. Pharm. Sci..

[63] ElShaer A., Khan S., Perumal D., Hanson P., Mohammed A.R. (2011). Use of Amino Acids as Counterions Improves the Solubility of the BCS II Model Drug, Indomethacin. Curr. Drug Deliv..

[64] Miller J.M., Dahan A., Gupta D., Varghese S., Amidon G.L. (2009). Quasi-Equilibrium Analysis of the Ion-Pair Mediated Membrane Transport of Low-Permeability Drugs. J. Control. Release.

[65] Samiei N., Mangas-Sanjuan V., González-Álvarez I., Foroutan M., Shafaati A., Zarghi A., Bermejo M. (2013). Ion-Pair Strategy for Enabling Amifostine Oral Absorption: Rat in Situ and in Vivo Experiments. Eur. J. Pharm. Sci..

[66] Miller J.M., Dahan A., Gupta D., Varghese S., Amidon G.L. (2010). Enabling the Intestinal Absorption of Highly Polar Antiviral Agents: Ion-Pair Facilitated Membrane Permeation of Zanamivir Heptyl Ester and Guanidino Oseltamivir. Mol. Pharm..

[67] Bashyal S., Seo J.E., Keum T., Noh G., Lamichhane S., Kim J.H., Kim C.H., Choi Y.W., Lee S. (2021). Facilitated Buccal Insulin Delivery via Hydrophobic Ion-Pairing Approach: In Vitro and Ex Vivo Evaluation. Int. J. Nanomed..

[68] Gamboa A., Schüßler N., Soto-Bustamante E., Romero-Hasler P., Meinel L., Morales J.O. (2020). Delivery of Ionizable Hydrophilic Drugs Based on Pharmaceutical Formulation of Ion Pairs and Ionic Liquids. Eur. J. Pharm. Biopharm..

[69] Iyire A., Alayedi M., Mohammed A.R. (2016). Pre-Formulation and Systematic Evaluation of Amino Acid Assisted Permeability of Insulin across in Vitro Buccal Cell Layers. Sci. Rep..

[70] Kang W.H., Van Nguyen H., Park C., Choi Y.W., Lee B.J. (2017). Modulation of Microenvironmental PH for Dual Release and Reduced in Vivo Gastrointestinal Bleeding of Aceclofenac Using Hydroxypropyl Methylcellulose-Based Bilayered Matrix Tablet. Eur. J. Pharm. Sci..

[71] Rawas-Qalaji M., Bafail R., Ahmed I.S., Uddin M.N., Nazzal S. (2021). Modulation of the Sublingual Microenvironment and PH-Dependent Transport Pathways to Enhance Atropine Sulfate Permeability for the Treatment of Organophosphates Poisoning. Int. J. Pharm..

[72] Kim J.C., Park E.J., Na D.H. (2022). Gastrointestinal Permeation Enhancers for the Development of Oral Peptide Pharmaceuticals. Pharmaceuticals.

